# Using Laser Tweezers For Manipulating Isolated Neurons In Vitro

**DOI:** 10.3791/911

**Published:** 2008-09-11

**Authors:** Robert Clarke, Jianfeng Wang, Ellen Townes-Anderson

**Affiliations:** Department of Neurology and Neuroscience, School of Medicine, University of Medicine and Dentistry of New Jersey

## Abstract

In this paper and video, we describe the protocols used in our laboratory to study the targeting preferences of regenerating cell processes of adult retinal neurons in vitro. Procedures for preparing retinal cell cultures start with the dissection, digestion and trituration of the retina, and end with the plating of isolated retinal cells on dishes made especially for use with laser tweezers. These dishes are divided into a cell adhesive half and a cell repellant half. The cell adhesive side is coated with a layer of Sal-1 antibodies, which provide a substrate upon which our cells grow. Other adhesive substrates could be used for other cell types. The cell repellant side is coated with a thin layer of poly-HEMA. The cells plated on the poly-HEMA side of the dish are trapped with the laser tweezers, transported and then placed adjacent to a cell on the Sal-1 side to create a pair. Formation of cell groups of any size should be possible with this technique. "Laser-tweezers-controlled micromanipulation" means that the investigator can choose which cells to move, and the desired distance between the cells can be standardized. Because the laser beam goes through transparent surfaces of the culture dish, cell selection and placement are done in an enclosed, sterile environment. Cells can be monitored by video time-lapse and used with any cell biological technique required. This technique may help investigations of cell-cell interactions.

**Figure:**
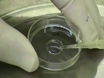


## Protocol

### Optical tweezers for manipulating isolated cells in culture

Trapping forces of optical tweezers are generated from the momentum of light (Ashkin, 1991; Ashkin et al., 1986). Although these forces easily trap cells in suspension, they are not able to move cells that adhere to a surface. Therefore, to reduce adhesion to the culture dish at the point where cells are trapped, the coverslip is coated with poly-2-hydroxyethylmethacrylate (poly-HEMA) (Sigma Chemical Co., St Louis, MO), a nontoxic compound with cell repellent properties previously employed for adhesion studies on endothelial cells (Folkman and Moscona, 1978). While poly-HEMA covers one half of the culture dish, the other half is coated with a substrate that supports cell growth. In our laboratory, Sal-1 is used as the substrate for culturing salamander retinal cells. The procedure for fabricating Sal-1 and poly-HEMA coated dishes is described below:

### Fabrication of culture dishes

Clean coverglasses (#1 VWR Scientific Inc., Media, PA) in acid. This coverglass was selected for its thickness of just 0.1mm, necessary for the high numerical aperture objectives required for focusing the laser.
To create a cell repellant area, dissolve 20 mg poly-HEMA (Sigma Chemical Co., St Louis, MO) in 1ml of 95% ethanol overnight at room temperature. Preferably, this solution should be used within 1 month of preparation.
Prop up the coverglasses at a near vertical position in a dust-free environment such as a hood.
Place one or two drops of poly-HEMA solution near the top of the coverglass from the plastic tip of a 200μl micropipette. The drops (about 50-100µl each) should be restricted to one half of the coverglass. The steep incline ensures the liquid flows down the surface of the coverglass to the bottom, providing a thin even coating of poly-HEMA. Allow the poly-HEMA coated coverglasses to dry in the hood for 30-60 minutes at room temperature.
Mark the poly-HEMA side of the coverglass with a fine tip pen close to the edge.
Drill a 1 cm hole in the bottom of a 35 mm culture dish.
Glue the poly-HEMA coated coverglass to the bottom of the culture dish with Sylgard 184 (Dow Corning Co., Midland, MI) so that the poly-HEMA coated side faces toward the inside of the dish. Ensure the edge of the poly-HEMA coating runs down the center of the hole. Allow the dishes to dry for 1-3 days at room temperature in a dust-free environment.
Scratch a line on the outside of the coverglass with a diamond-tip pen following the edge of the poly-HEMA coating. Then, scratch two crossing lines at right angles to it approximately 2mm apart. The two points of intersection serve as reference or fiducial points to be used for reproducing the same orientation of the culture dish on the microscope stage.
Sterilize the dishes under ultraviolet light overnight.
To create a cell-adherent half of the culture dish, coat the non poly-HEMA half with Sal-1, which contains a mouse anti-salamander antibody raised against retinal cell membranes (generously provided by Dr. Peter MacLeish, Morehouse School of Medicine, Atlanta, GA; see MacLeish et al. (1983)).  First, coat the Sal-1 side with approximately 100µl of 0.1 mg/ml sterile goat anti-mouse IgG antibody (Boehringer Mannheim Corporation, Indianapolis IN), taking care not to spill the liquid over onto the poly-HEMA side. Leave for three hours, then remove it. Wash the same area with sterile Ringer’s solution once or twice, then place 100µl Sal-1 supernatant, taking care not to spill the liquid into the poly-HEMA side of the dish. The first antibody ensures Sal-1 is coated at a known concentration.
Incubate the dishes with the Sal-1 overnight.
Remove the Sal-1 antibody solution and rinse the area with sterile Ringer’s solution. Introduce 2 ml medium to the dish just prior to cell plating.

The composition of the amphibian Ringer’s solution, serum-free amphibian medium, and Ringer’s solution for enzyme digestion are found in MacLeish and Townes-Anderson (1988) and Mandell et al. (1993).

### Preparation of cell cultures

The cells used in this video were derived from light-adapted, adult, aquatic-phase tiger salamanders (Ambystoma tigrinum), measuring 17–22 cm in length (Charles Sullivan Inc., Nashville, TN), maintained at 5°C on a 12 h:12 h light-dark cycle. The protocols were approved by the Institutional Animal Care and Use Committee (IACUC) at the University of Medicine and Dentistry of New Jersey in strict accordance with the guidelines from the National Institutes of Health. The procedures for preparing retinal cell cultures are described by Nachman-Clewner and Townes-Anderson (1996) and are as follows:

Decapitate and pith the animals. Remove the eyes and dissect out the corneas and iris. The retinas should detach slightly within the eyecup. By placing a curved forceps underneath the retina, scoop out the retinas. It is not important if the lens is attached to the retina at this stage.
Digest the retinas in 14 U/ml papain (Worthington, Freehold, NJ) in salamander Ringer's solution for 40 mins.
Prior to use, the papain Ringer’s solution should be bubbled with 95% O_2_ / 5% CO_2_  gas for 30 mins., then sterilized by passage through a 0.22 micron filter (Millipore, Carrigtwohill, Ireland).
Rinse the retinas with sterile salamander Ringer’s solution three times.
Remove the lenses from the Ringer’s solution, then gently triturate the retinal tissue with a 3 mm bore pipette to yield a cell suspension.

### Laser tweezers manipulation of isolated neurons

Place the culture dish on the microscope stage, orienting the dish by aligning a mark on the dish with a reference point on the stage. Save the coordinates of the fiducial points in the computer.
Plate cells onto both Sal-1 and poly-HEMA halves of the dish.
Allow the cells to settle for approximately 20 mins, which is long enough for cells on the adherent side of the dish to attach to the antibody substrate.
Select a cell on the Sal-1 adherent side of the dish. Mark the x and y stage coordinates of a position close to the cell. In our case, the point was approximately 10µm from the primary dendrites of the selected cell. Save coordinates in the computer.
Select a second neuron, in our case a photoreceptor, on the poly-HEMA side of the dish and use the laser tweezer tool to trap it.  Trapping can be achieved over a broad range of power levels. However, we set the power of the laser at 10%–20%, which is low enough to avoid trapping debris along with the cell, but sufficient to transport the cell through the medium.
While holding the cell in the trap, lower the stage so that the cell is well above the surface of the culture dish and above any attached neurons. Move the stage under computer control to bring the cell to the previously saved x and y coordinates on the Sal-1 side. The stage movement was set at 8–20µm/s, but maximum speed should be empirically determined. On arrival at the destination point on the Sal-1 side, raise the stage to bring the trapped cell to the surface of the dish. Cell placement can be made with an accuracy of a few microns. Allow the cell to adhere to the Sal-1 substrate.
Obtaining digitized images of both cells in the pair before and after pair formation provides a useful record.
Maintain the cell pairs in an incubator. For salamander cells, place the dishes in a humidified chamber at 10°C. Cells can be monitored by video time lapse and used with any cell biological technique required.

## Discussion

Light has momentum, and when a light ray is refracted as it passes through a cell, a force is required to change the direction of the momentum. Because of the law of conservation of momentum, a force in the opposite direction must, in turn, react back on a cell. Ashkin (1991) showed that the force generated by a laser beam focused by a microscope objective lens will move a cell toward the center of focus. Even though a laser beam generates only a few piconewtons of force, this force is sufficient to pull a cell through medium. A near infrared wavelength that is poorly absorbed by water is used to avoid damage to the cell (Liu et al 1995). The laser-tweezer workstation used in our laboratory (Cell Robotics Inc., Albuquerque NM)  uses a 1 W, continuous wave diode laser of 980nm wavelength. The laser light is transmitted to the cells via a 40x oil immersion plan neofluor objective lens of high numerical aperture (N.A.1.3) (Carl Zeiss Inc.) which focuses the beam at the same focal plane as the microscope.

Laser-tweezers-controlled micromanipulation presents a number of advantages over studies on randomly plated cells. For example, cells can be placed at a desired distance from one another which can be standardized for all manipulated cells. In addition, the laser beam passes through the transparent surface of the culture dish and, therefore, cell manipulation is done in an enclosed, sterile environment. The very small forces generated by the laser places a limit on what can be trapped. In our experience, adhesion and cell shape are the most important factors limiting successful trapping. In general, we trap isolated cells of 10-15μm in diameter and transport them distances of several millimeters across the culture dish to their destination. It is possible to move larger cells than these, e.g., Muller glial cells, which are 20-40μm in length and are the largest cells in our cultures.

Because trapping cells is a challenge when the cells adhere to the glass bottom of the culture dish and to each other, developing a cell-repellant surface on the coverglass using poly-HEMA proved invaluable to our success in manipulating cells. Even so, the cells on the poly-HEMA surface eventually became stickier and difficult to manipulate in cultures more than 1-2 hours old. In older cultures, the common practice in our lab was to try to pick up the cells with the laser using the maximum power setting, then turn the power down to transport it.

The ease with which small objects can be trapped in the laser means that debris can be pulled into the trap from distances well outside the cell. This proved to be a problem during the trapping of our cells. This can be avoided in most instances by turning down the power of the laser to the minimum possible that can hold the cell within the trap. The speed at which cells can be transported in the trap is limited by the viscosity of the medium. We normally set the speed at around 8-20 μm/second which was found to be within a safe range for transport.

The possibility that the laser has detrimental effects on the cells held in the trap must be considered. In our retinal cultures, we encounter dark objects such as pigment cells which are destroyed when captured in the laser beam. Clearly, therefore, these cells cannot be studied using laser tweezers. However, in previous studies on retinal neurons and photoreceptors carried out in our laboratory, we found no difference in neuritic growth and capacity to form connections between tweezer-manipulated cells and non-manipulated cells (Townes-Anderson et al 1998; Clarke et al 2008).
